# Trap-Controlled Conduction and Metal–Insulator Transition in Superconducting Cuprate Memristors

**DOI:** 10.1021/acsaelm.5c02017

**Published:** 2026-01-16

**Authors:** Thomas Günkel, Enrique Miranda, Lluís Balcells, Narcís Mestres, Anna Palau, Jordi Suñé

**Affiliations:** † 54449 Institut de Ciència de Materials de Barcelona (ICMAB-CSIC), Campus de Bellaterra, 08193 Bellaterra, Barcelona, Spain; ‡ Departament d’Enginyeria Electrònica, Universitat Autònoma de Barcelona, 08193 Bellaterra, Barcelona, Spain

**Keywords:** cryogenic memristor, resistive switching, conduction mechanisms, trap-controlled space-charge-limited conduction, high-temperature superconductor, metal−insulator transition, neuromorphic computing

## Abstract

Memristive devices based on high-temperature superconducting cuprates offer promising routes for neuromorphic computing, yet the microscopic mechanisms governing their resistive switching remain unclear. Here we investigate YBa_2_Cu_3_O_7−δ_ (YBCO) memristors across 80–300 K, revealing robust bipolar switching between high- and low-resistance states with temperature-independent SET and RESET voltages. Current–voltage analysis shows both states follow trap-controlled space-charge-limited conduction, modulated by shallow and deep trap states at an oxygen-deficient interfacial YBCO layer. A key enabler of this behavior is the formation of a deoxygenated layer beneath the top contact, which acts as a dynamic trap region and allows electrostatic control over a field-induced metal–insulator transition. We propose a dual-trap model where deep traps linked to CuO chain fragmentation stabilize a field-induced metal–insulator transition, enabling nonvolatile switching. These insights elucidate the role of trap dynamics in cuprate memristors and highlight their potential for cryogenic neuromorphic platforms compatible with superconducting computing architectures.

## Graphical abstract

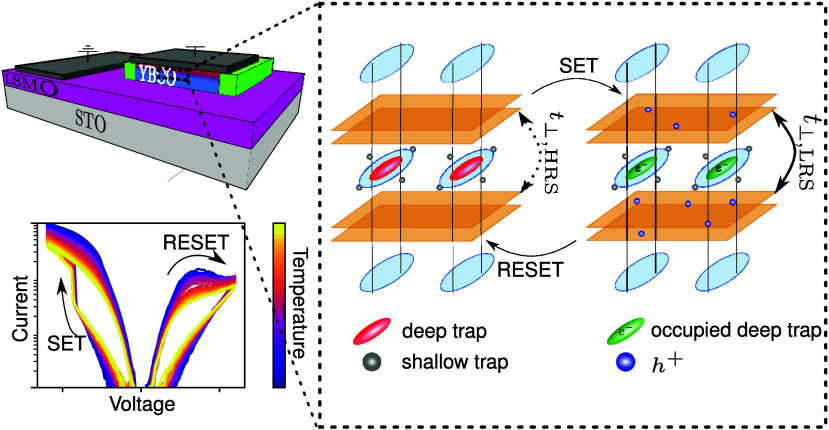

## Introduction

The exponential growth of data generation and the limitations of conventional computing architectures have intensified the search for advanced memory technologies that offer high performance, energy efficiency, and scalability.^1^ Among different emerging candidates, memristive devices first described theoretically in 1971,^2^ are gaining growing interest due to their applications in the field of neuromorphic computing or multilevel memories,^3,4^ which might overcome the limitations of the conventional von Neumann architecture.^5^ Memristors have evolved from research prototypes into commercially available technologies with applications in memory and computing, as reviewed in.^6^ These devices exhibit exceptionally low energy consumption, reaching values as low as 500 fJ per switching event and approximately 1 fJ per read operation.^7,8^ Furthermore, their multistate retention capability enables in-memory computing,^9^ which has been demonstrated in several prototype RRAM-based chips integrating data-conversion functions.^10,11^ After the first experimental realization of a memristive device^12^ a wide range of materials and switching mechanisms have been investigated.^13^ Among these, Mott insulators have attracted particular interest due to their capacity to exhibit electrically tunable metal–insulator transitions (MITs) driven by carrier doping modulation.^14^ This tunability is especially interesting in the case of high-temperature superconducting cuprates, which not only allow transitions between insulating and metallic states but also provide access to a variety of electronic phases—including superconductivity—across their temperature–doping phase diagram.^15^ The ability to reversibly control these phase transitions via external stimuli, such as electric fields, makes cuprates ideal candidates for multifunctional cryogenic devices. In particular, their integration into superconducting neuromorphic architectures offers a promising route toward energy-efficient, low-temperature computing platforms.^16,17^ These systems are especially relevant in the context of quantum technologies, where cryogenic compatibility is essential. By enabling local, low-power processing near quantum processors, superconducting neuromorphic devices could help bridge the gap between classical neuromorphic computing and quantum information processing.^18–20^ In this context, several studies have focused on electrically modulating the electronic properties of archetypal cuprate superconductors such as YBa_2_Cu_3_O_7−δ_ (YBCO) and Bi_2_Sr_2_CaCu_2_O_8+δ_ (BSCCO) through field-effect gating techniques.^16,17,21–24^ A common structural feature of these materials is the presence of CuO_2_-planes, which become hole-doped via oxygen incorporation and are responsible for their superconducting behavior.^25^ In a recent study, we demonstrated that memory cells composed of superconducting YBCO active layers, integrated with La_0.3_Sr_0.7_MnO_3_ (LSMO) as the bottom electrode and gold as the top contact, exhibit two distinct resistive switching mechanisms with opposite polarities: one driven by oxygen-ion migration dominant at high temperatures, and another governed by charge carrier trapping and detrapping processes, which becomes significantly enhanced at cryogenic temperatures.^17^ A crucial element enabling the latter mechanism is the formation of an oxygen-depleted interfacial layer at the Au/YBCO interface during fabrication. This layer acts as a tunable region for charge trapping, facilitating nonvolatile resistive switching effects through an electrostatically induced metal–insulator transition. In this work, we investigate the relevant conduction mechanisms in these YBCO-based memristive devices to better understand the microscopic factors that determine the resistive states. By systematically analizing the temperature-dependent current–voltage characteristics we find that both the HRS and LRS are consistent with space-charge-limited conduction (SCLC) governed by trap states located at the oxygen depleted Au/YBCO interface. Understanding these mechanisms is important not only for revealing the fundamental physics of the memristor cell but also for improving its performance.

## Results and Discussion

The devices studied in this work are based on superconducting YBCO thin films with thicknesses of 30, 50 and 100 nm, deposited on SrTiO_3_ (STO) substrates. A 40 nm thick ferromagnetic LSMO layer is used as a buffer and simultaneously serves as the bottom electrode. The top electrodes consist of 100 nm thick layers of gold. To define the active regions, square-shaped YBCO islands with lateral dimensions of 80 × 80 μm^2^ were patterned using standard photolithography. These islands were partially coated with a 30 nm thick insulating layer of Al_2_O_3_ to prevent electrical shorting between the gold and LSMO electrodes. A sketch of the devices is presented in Figure 1a.

**Figure 1:**
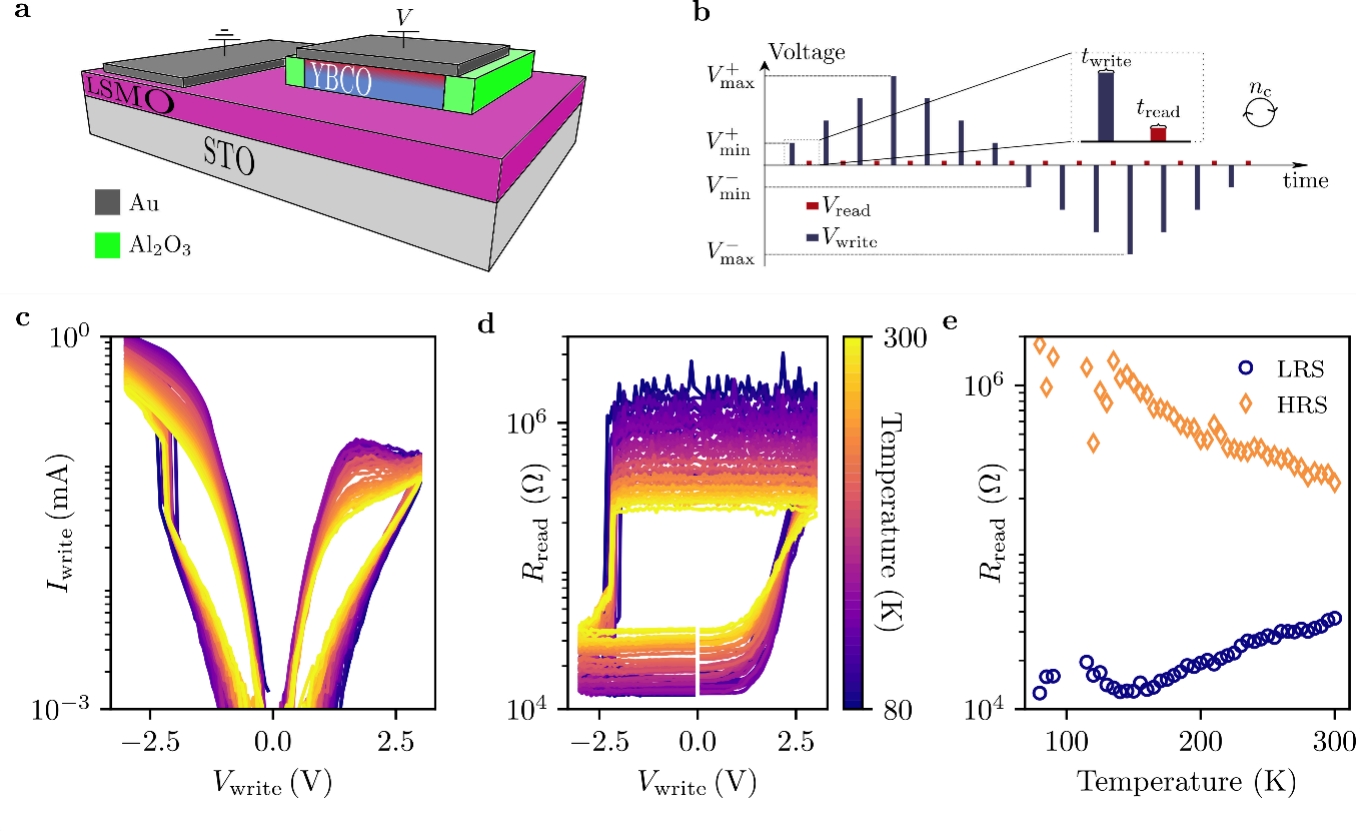
a) Schematic layout of the devices. b) Schematic representation of the pulse train used to obtain the resistance hysteresis loops, consisting on alternating writing and reading pulses. For all measurements parameters are kept constant as *t*_write_ = 0. 3 ms, *t*_read_ = 0. 3 ms, *V*_read_ = −1 V, *V*_min_^+^ = −*V*_min_^–^ = 0.1 V and *n*_c_ = 3. c) Temperature dependence of the current–voltage characteristics measured under positive and negative voltage pulses for a device of 30 nm. d) Resistance hysteresis lops obtained at different temperatures for the same device. The resistance dispersion observed at HRS for low temperatures is associated with measurement resolution constraints. e) Temperature dependence of the resistances in the HRS and LRS obtained with the loops shown in d).

Top-bottom electric measurements were conducted across a broad temperature range of 80–300 K using alternating writing and reading pulses, which allow to evaluate the *IV* characteristics, plotting *I*_write_ vs *V*_write_, as well as the resistance hysteresis, obtained through *I*_read_ vs *V*_read._ Here *I*_write_ denotes the current measured with a writing voltage, *V*_write_, and *I*_read_ is the current measured with the reading voltage, *V*_read_). To avoid altering the device state, the reading voltage is maintained below a threshold. A schematic of this method is provided in Figure 1b. Figure 1c illustrates the *IV* characteristics at various temperatures for a device with a thickness of 30 nm. It can be seen that the I–V curves exhibit hysteresis, with a RESET transition from a low-resistance state (LRS) to a high-resistance state (HRS) under positive bias and a SET transition from the HRS to the LRS under negative bias. The resulting resistance hysteresis at different temperatures, measured with a reading pulse of *V*_read_ = −1 *V* is depicted in Figure 1d. It is noteworthy that the voltage required for the SET transition remains constant, *V*_SET_ ≈ −2.2V across the entire temperature range. A similar, temperature-independent, behavior is observed for the RESET transition from LRS to HRS. However, this transition occurs more gradually within a voltage range of ≈1–3 V. The current–voltage (*IV*) characteristics and resistance hysteresis loops for devices of 50 and 100 nm are presented in Figure S1 of the Supporting Information. The robustness of the switching effect across the entire temperature range rules out ion migration as the underlying mechanism. To assess cycle-to-cycle variability, three consecutive switching loops were recorded at each temperature, as shown in Supplementary Figure S3, demonstrating good reproducibility of the resistive states. Importantly, these measurements extend to temperatures below the superconducting transition of the YBCO thin films (*T*_*c*_ ≈ 80 K), as determined by SQUID magnetometry, confirming that the switching behavior persists in the superconducting regime. Furthermore, preliminary tests indicate good retention at both 77 and 150 K, with cryogenic conditions being more favorable for stable operation, as discussed in our previous work.^17^ Figure 1e shows the temperature dependence of the resistance at the HRS and LRS. In the LRS, the resistance decreases upon cooling, indicative of metallic behavior, whereas in the HRS, it increases with decreasing temperature, consistent with a field-induced metal–insulator transition.

Conduction mechanisms in resistive switching devices are typically classified into interface-limited and bulk-limited processes. Interface-limited mechanisms, such as Schottky barrier modulation, thermionic emission, and tunnelling, are governed by the properties of the electrode–material interface. In contrast, bulk-limited mechanisms involve charge transport through the active layer and include ohmic conduction, Poole–Frenkel emission, or space-charge-limited conduction, often influenced by trap-assisted transport or hopping between localized states.^26^ Analyzing the temperature and voltage dependence of the current is the usual way to discriminate between these conduction mechanisms. To exclude filamentary conduction, we performed measurements on devices with different areas, presented in the Supporting Information (Figure S2), which show that both HRS and LRS depend on the device area. Figure 2a,b and c,d presents the log–log *I*–*V* plots obtained for a 50 nm device at 30 and 100 K during the SET and RESET transitions, respectively. The fitted curves in Figure 2 reveal a power-law dependence of the form *I* ∝ *V* ^*m*^, which is characteristic of space-charge-limited conduction (SCLC), a mechanism commonly observed in semiconducting materials.^26–30^ This power-law scaling is observed in both the HRS and LRS suggesting that bulk-limited transport dominates in both regimes. Further support to this interpretation comes from the symmetry of the *IV* characteristics in both resistive states in Figure 1c. This symmetry is not expected for interface-limited mechanisms. Although there is some dispersion in the reported values for the work function of the involved materials, the hypothesis of no interface Shottky barrier is compatible with the literature results. For instance, 5.1 eV have been reported for Au,^31^ 4.9 eV for LSMO^32–34^ and 5 eV for YBCO.^35,36^ With these values, no significant interface potential barriers are expected in the Au/YBCO/LSMO/Au structure and Ohmic contacts yielding symmetric conduction are a reasonable assumption. Previous studies on YBCO systems primarily associated resistive switching with mechanisms linked to oxygen diffusion. For example, the electric behavior of Pt/YBCO structures with different oxygen concentrations could be modeled using a Poole–Frenkel framework, demonstrating that oxygen vacancies form potential wells that trap carriers and strongly influence conduction.^37^ In related work on Au/YBCO junctions, resistive switching was attributed to variable-range hopping processes modulated by the concentration of oxygen vacancies.^38^ Other authors have explicitly connected resistive switching to interfacial redox reactions involving oxygen,^39,40^ or to bulk mechanisms in transistor-like structures that rely on oxygen mobility.^16,41^ Collectively, these studies emphasize the central role of oxygen diffusion in previously reported switching phenomena. In contrast, the experimental evidence presented in this work indicates that such oxygen-related mechanisms do not account for our observations. Instead, our results are consistent with a charge-diffusion mechanism, where resistive switching arises from the trapping and detrapping of electrons at oxygen-vacancy sites, without requiring vacancy migration. Furthermore, the robustness of the switching effect across the whole temperature range, together with the temperature-independent switching voltage, rules out ion (oxygen) migration as the dominant mechanism.

**Figure 2:**
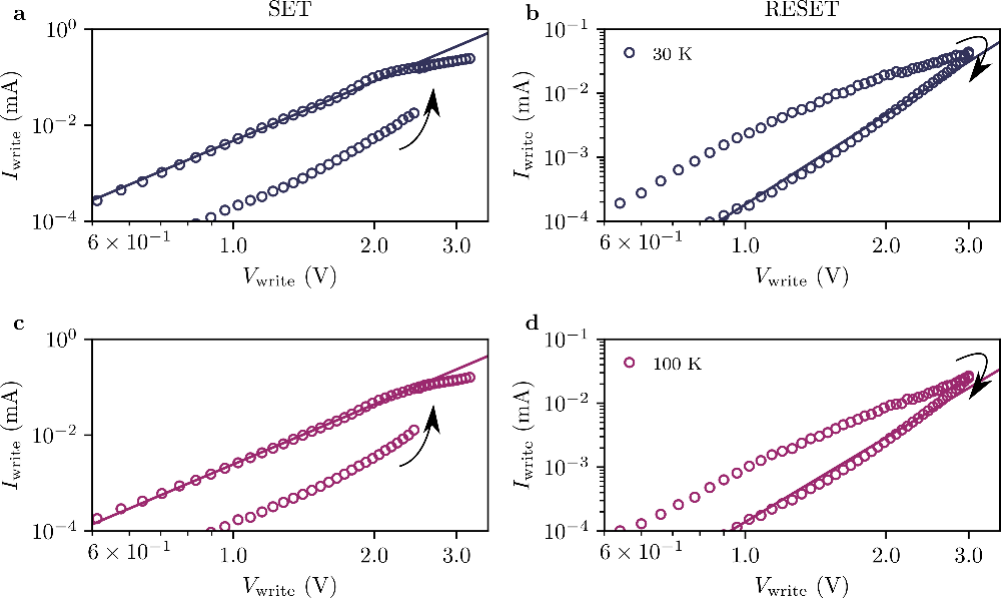
Current–voltage characteristics of the a,c) SET and b,d) RESET transitions obtained for a 50 nm device at 30 K (a,b) and 100 K (c,d). Solid lines are fits to a power law dependence, *I* ∝ *V* ^*m*^.

To further clarify the transport mechanisms, Figure 3a shows the *IV* characteristics measured for a device of 50 nm thickness in the HRS at temperatures ranging from 80K to 300 K, measured in steps of 5 K. Figure S4 in the Supporting Information shows the equivalent data obtained for devices of 30 and 100 nm. The best fit of the data is provided by a power law *I* ∝ *V* ^*m*^, which is consistent with SCLC. The corresponding power-law exponents are presented in Figure 3b. The analysis using the SCLC model is one of the experimental methods for the detection of charge-trap states in disordered semiconductors.^29^ In the case of a trap-free semiconductor the current–voltage relationship follows the Mott–Gurney law, which predicts a quadratic dependence of the current on the voltage,^42,43^ provided as

1 $$J=\frac{9}{8}\epsilon_{0}\epsilon_{s}\mu \frac{V^{2}}{L^{3}}$$

where ϵ_s_ is the permittivity of the material, ϵ_0_ is the vacuum permittivity, μ is the carrier mobility and *L* the thickness of the active layer. In SCLC with traps, the nature of the trap energy distribution significantly influences the current–voltage (*I*–*V*) characteristics and the power law exponent *m* > 2.^28,29,44^

**Figure 3:**
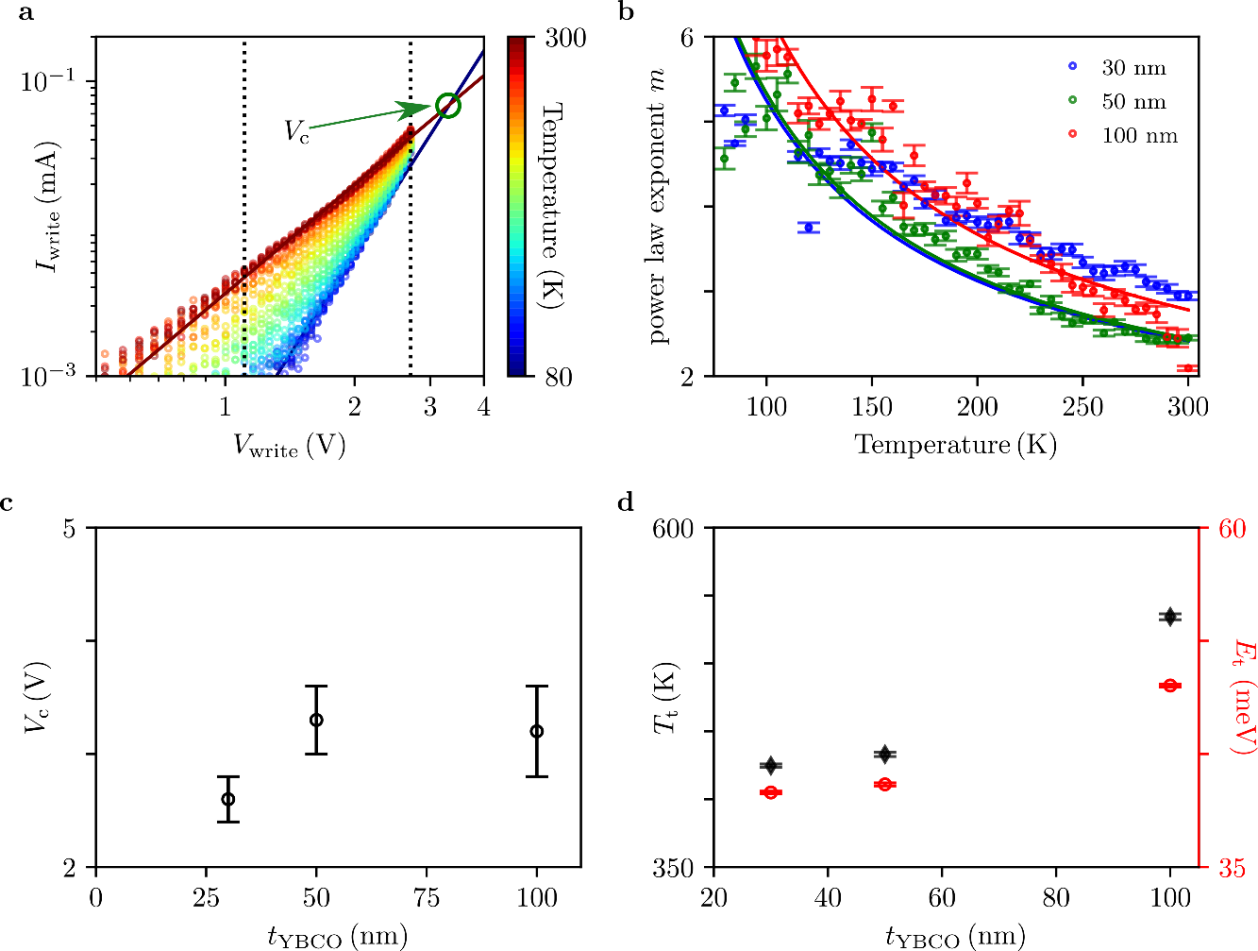
a) Log–log plot of the current–voltage characteristics of the HRS obtained at different temperatures for a device of 50 nm. The solid lines show power law fits for the experimental data at 300 and 80 K. The dashed lines show the voltage range used to fit the power law dependence. b) Temperature dependence of the power law exponents extracted for samples of different thicknesses. Solid lines show the fits obtained to a *m* = 1 + *T*_t_/*T* dependence. c) Critical voltage *V*_c_ obtained for devices of different thickness. The error bar of *V*_c_ is estimated by a graphical extraction illustrated in the Supporting Information. d) Characteristic temperature, *T*_t_ (black diamonds), and energy, *E*_t_ (red circles) extracted from the fits shown in panel b.

In the HRS and LRS, the *I*–*V* curves can be well described by a power-law dependence with *m* > 2, indicating that trap states play an active role in charge transport. In the presence of trap states that are exponentially distributed in energy with a density *d*_t_(*E*) given by^30^

2 $$d_{t}\left(E\right)=\frac{H_{t}}{E_{t}}\exp\left(-\frac{E}{E_{t}}\right)$$

were *H*_t_ is total trap density and *E*_*t*_ the characteristic energy of the trap distribution. By substituting *E* = *k*_b_ *T* and *E*_t_ = *k*_b_ *T*_t_, the distribution can be expressed in terms of the characteristic temperature, *T*_t_, which reflects the steepness of the trap energy profile. A small *T*_t_ corresponds to a sharply varying trap distribution, while a large *T*_t_ indicates a more gradual variation.^27,28,44^ In this model the current voltage characteristics is given by the Mark–Helfrich equation,^30^

3 $$J=N_{0}\mu q^{1-l}\left(\frac{\epsilon_{0}\epsilon_{s}l}{H_{t}\left(l+1\right)}\right){\left(\frac{2l+1}{l+1}\right)}^{l+1}\left(\frac{V^{l+1}}{L^{2l+1}}\right)$$

with *N*_0_ being the effective density of states and the elementary charge *q*. The exponent *m* = *l* + 1 provides a direct measure of the trap distribution’s influence on charge transport.

Figure 3b shows the temperature dependence of the power law exponent *m* obtained for devices of different thickness in the HRS, which follows the expected relation *m* = 1 + *T*_t_/*T* for every device. Another key feature emerging from the model is the presence of a critical voltage, *V*_*c*_, at which the current becomes temperature independent. Analytically the critical voltage can be derived from the Arrhenius form of eq 3,^28^ which is given by

4 $$J=\left(\frac{\mu N_{0}qV}{2L}\right)f\left(l\right)\exp\left[-\frac{E_{t}}{k_{b}T}\ln\left(\frac{qH_{t}L^{2}}{2\epsilon_{0}\epsilon_{s}V}\right)\right]$$

here *f*(*l*) denotes a function which only depends on *l*. The critical voltage is defined as the voltage where the activation energy

5 $$E_{a}=-\frac{E_{t}}{k_{b}}\ln\left(\frac{qH_{t}L^{2}}{2\epsilon_{0}\epsilon_{s}V}\right)$$

becomes zero. Thus, the critical voltage is given by

6 $$V_{c}=\frac{qH_{t}L^{2}}{2\epsilon_{0}\epsilon_{s}}$$

Experimentally this voltage is obtained by extrapolating the fitted *IV* dependencies to higher voltages as illustrated in Figure 3a. This behavior implies that the activation energy for conduction vanishes at *V*_c_, consistent with a transition from thermally activated trap-assisted transport to field-driven conduction. To further elucidate the trap states, we analyze the temperature-dependent *IV* characteristics to extract the critical voltage in [Fig fig3]c and the characteristic temperature in [Fig fig3]d for each device. Figure S4 in the Supporting Information illustrates, how *V*_c_ was extracted for every characterized device. Assuming that the trap density and the dielectric constant of the active layer are the same for all devices, one can conclude that not the entire thickness of the YBCO thin film, *L*, participates in the resistive switching; otherwise, due to eq 6 there would be quadratic dependence of the critical voltage on L. In other words, one may assume that the active layer has nearly the same thickness in all measured devices. An exact quantification of the trap density from eq 6, however, is only possible if both the precise thickness of the active layer and its permittivity are known. For the devices analyzed here, such an extraction is not possible, since all properties of YBa_2_Cu_3_O_7−δ_, including the permittivity, strongly depend on the oxygen stoichiometry and on the temperature. Reported dielectric constants vary widely, from ϵ_s_ = 2 up to 10^8^, depending on the aforementioned conditions.^45–48^ To estimate the trap density, we therefore assume a dielectric constant of ϵ_s_ = 1000 and an active deoxygenated YBCO layer thickness of 10 nm.^45^ Using these values and the critical voltage *V*_*c*_ ≈ 3 V extracted from Figure 3c, we obtain a trap density of approximately 0.57 per unit cell. Assuming that each oxygen vacancy acts as a single trapping center, this analysis suggests that the active YBCO in our memristive devices is highly deoxygenated, with a stoichiometry of about δ ≈ 0.6. This estimate is only meant to support the notion that deoxygenated YBCO is indeed responsible for resistive switching in our memristive cells. Nevertheless, it should be noted that the actual situation is far more complex, as the origin of the shallow traps could be of a different kind, and is addressed in more detail in the discussion. To conclude the analysis of the HRS, the characteristic energy *E*_t_ is calculated from the fitted *T*_t_, and plotted in Figure 3d. The extracted trap energies of around 40–50 meV are responsible for the conduction mechanism but are too shallow to account for the nonvolatile behavior of the devices.

The consistency of these results across device geometries and temperatures provides strong evidence for a trap-controlled SCLC mechanism in the HRS. Thus, it is natural to study the LRS within the same theoretical framework. As shown in Figure 4a, the current–voltage characteristics can be fitted with a power-law exponent at all temperatures. However, although a power-law dependence is observed, the obtained exponent *m* ≈ 3 remains nearly constant across the measured temperature range, as seen in Figure 4b, suggesting a trap-filled limit (TFL) regime in which most trap states are saturated and the current is dominated by free-carrier transport. The deviation from the ideal Mott–Gurney law—which predicts *m* = 2 for trap-free SCLC—indicates the presence of residual shallow traps or a nonuniform trap distribution. These traps may not be fully filled or could be distributed in energy, thereby modifying the current–voltage relationship. Interestingly, applying the same method used for the HRS to fit the experimental data above 200 K one obtains a characteristic energy of the same order of magnitude, *E*_t_ ≈ 40 meV. This suggests that the conduction is still governed by the same kind of traps in this temperature regime, but the conduction mechanism evolves as the devices are cooled down. To investigate alternative mechanisms underlying LRS behavior, the experimental data were compared with several existing models.^26^ However, none of these models adequately explained the observed *IV* characteristics in the LRS across a broad voltage range. One plausible mechanism is trap-assisted tunnelling (TAT), which provides a good theoretical fit for the 30 nm device (See Supporting Information Figure S5).

**Figure 4:**
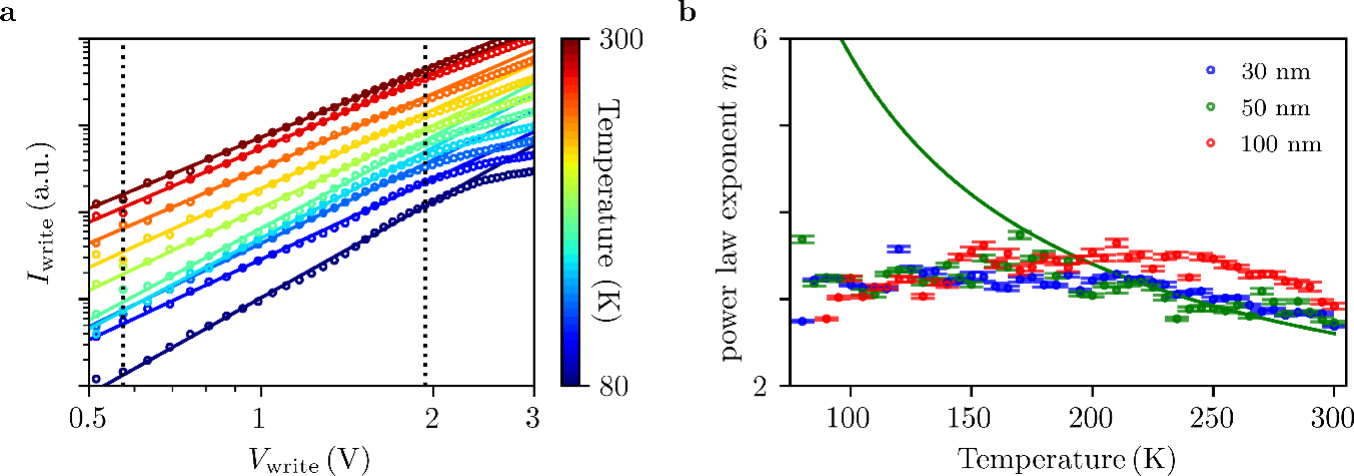
a) Log–log plot of the current–voltage characteristics of the HRS obtained at different temperatures for a device of 50 nm. For clarity the *IV* curves are vertically offset along the current axis. The solid lines show power law fits for the experimental data and the dashed lines show the voltage range used to fit the power law dependence. b) Temperature dependence of the power law exponents extracted for samples of different thicknesses. Solid lines show a fit obtained to a *m* = 1 + *T*_t_/*T* dependence.

A notable advantage of these memristor devices, particularly for neuromorphic and memory applications, is their ability to stabilize multiple resistance states during the RESET transition.^17^ This enables a study of the evolution of the *IV* characteristics for intermediate states. Figure 5a shows the evolution of the *IV* characteristics for different RESET voltages, measured in a 30 nm-thick device at 77 K. It is evident that higher RESET voltages result in a larger window of the *IV* characteristics. All *IV* curves obtained after the RESET transition again follow a power-law dependence, with an exponent gradually increasing from *m* ≈ 3 in the LRS to about *m* ≈ 4.5 in the highest resistive state. This development is shown in Figure 5b. It indicates that the traps governing the conduction mechanism become more active in higher resistive states. The fits are shown in Supporting Information Figure S6.

**Figure 5:**
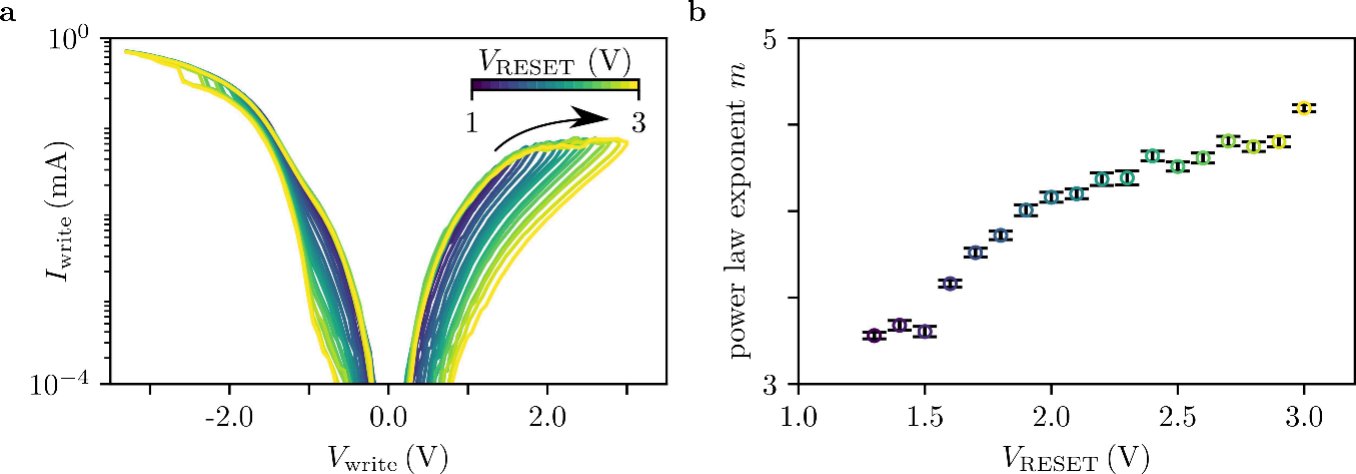
a) Current–voltage characteristics obtained for a 30 nm device at 77 K, after performing RESET transitions with steadily increasing voltages in the range of *V*_reset_ 1 V → 3 V, indicated by the color code of the label bar. To ensure saturation in the LRS, the negative voltage was swept up to *V*_max_^–^ = −3 V for each recorded minor loop. b) Evolution of the power law dependence with the applied RESET voltage.

Overall, the distinct temperature dependencies and power-law exponents observed in the HRS and LRS underline the central role of trap dynamics and electric field effects in governing the resistive switching and conduction mechanism of YBCO-based memristive devices. As discussed in our previous work,^17^ we assume that the resistive switching arises from a field-induced MIT intrinsically linked to a charge trapping and detrapping process. The epitaxial lattice of YBCO, as illustrated in Figure 6a, consists of CuO-chains and CuO_2_-planes. Superconductivity in the CuO_2_-planes emerges upon oxygen doping of the CuO-chains, where inserted oxygen atoms remove electrons from the planes, leading to effective hole doping. This doping can be externally modulated via oxygen field-induced oxygen migration,^37,41^ electrostatic doping,^15^ or photodoping.^49^ In the case of photodoping, photoexcitation generates electron–hole pairs, followed by electron trapping and hole promotion in the CuO_2_-planes, effectively mimicking chemical doping. For nonvolatile switching, trapped electrons are stabilized by charged defects such as oxygen vacancies in fragmented CuO-chains.^50,51^ In this way, nonvolatile transitions from insulating to superconducting states in BSCCO structures may be explained by carrier injection.^21^ To better understand these phenomena, it is useful to recall that superconducting cuprates are Mott insulators, where the interplay between charge-carrier localization and delocalization governs the transitions between insulating and metallic phases. In the underdoped regime, strong electron correlations lead to carrier localization, resulting in a Coulomb repulsion energy *U* that exceeds the kinetic energy of electrons. This interaction splits the half-filled conduction band into two distinct sub-bands: the lower Hubbard band (LHB), which is fully occupied, and the upper Hubbard band (UHB), which is empty.^52^ The Fermi level lies within the gap between these bands, giving rise to insulating behavior despite a nominally metallic band structure. More specifically, YBCO is a charge-transfer Mott insulator, where the gap lies between the O 2p band and the Cu 3d UHB, and the charge transfer energy Δ is smaller than the repulsion energy *U* (see Figure 6b). In its undoped state, YBCO exhibits a charge-transfer gap of approximately 1.5–2 eV,^53^ which is consistent with the voltage required to induce the abrupt transition from HRS to LRS. Doping, whether chemical, photonic, or electrostatic, introduces holes primarily into the O 2p band, modifying the electronic structure and enabling carrier delocalization. This screens Coulomb interactions and collapses the Mott gap, facilitating a transition to a metallic state within the CuO_2_-planes (see Figure 6c). While the collapse of the Mott (or charge-transfer) gap upon doping explains the emergence of metallic conduction within the CuO_2_-planes, supercondcucting below *T*_*c*_, out-of-plane (*c*-axis) transport in YBCO is additionally governed by weak interlayer coupling (*t*_⊥_) between planes of different unit cells (see Figure 6d,e). In the underdoped state, strong correlations suppress coherent interlayer motion, leading to insulating-like *c*-axis transport. Upon doping, the enhanced carrier delocalization and screening not only promote in-plane metallicity but also increase the effectiveness of interlayer coupling, thereby modifying the *c*-axis conductivity.^54–56^ Based on the former discussion, we provide an enhanced physical picture of our earlier work.^17^ In the HRS, the devices contain an underdoped YBCO layer with broken CuO-chains beneath the top electrode, as schematically shown in Figure 6d. In this situation, the interlayer coupling *t*_⊥,HRS_ between the CuO_2_ planes is weak and the devices exhibit an insulating-like behavior. This defect-rich layer can trap injected electrons once a certain threshold voltage *V*_set_ ≈ 2–2.5 V, sufficient to induce a metal–insulator transition, is exceeded. As illustrated in Figure 6e, electron trapping may result in charge redistribution, thereby increasing the effective hole concentration in the CuO_2_-planes. This induces a nonvolatile transition to the metallic state which in turn enhances the interlayer coupling *t*_⊥,LRS_ and the *c*-axis conductivity. The trapped electrons within the CuO-chains stabilize the doped LRS, preventing relaxation upon bias removal.^50,51^

**Figure 6:**
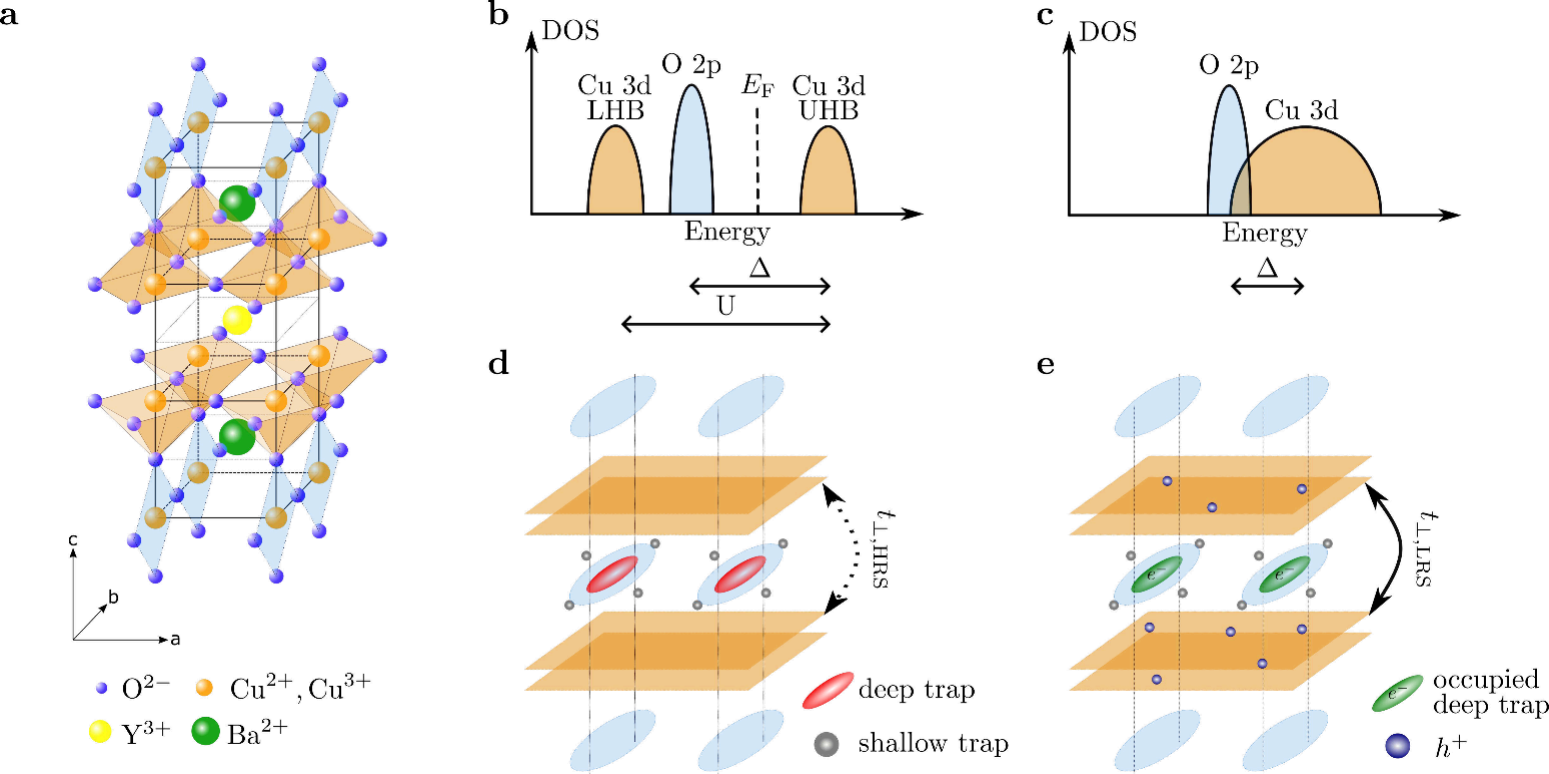
a) Schematic illustration of the YBCO crystal structure. The respective ions and their color code are indicated in the legend. b,c) Evolution of the electronic band diagram during the metal–insulator transition: (b) insulating state with Hubbard band splitting and (c) metallic state following gap collapse. d, e) Schematic representation of deep and shallow trap filling at the HRS and LRS, respectively. Light blue ellipses represent CuO chains, and orange sheets denote CuO_2_-planes. *t*_⊥,HRS_ and *t*_⊥,LRS_ indicate the coupling between the planes in the HRS and LRS, respectively.

To account for the observed electrical behavior, we propose a model involving two distinct types of trapping centers within the system. (i) Shallow-energy traps, likely associated with point defects such as oxygen vacancies or more exotic states such as polarons,^57^ exhibit an exponential energy distribution with a low activation energy of approximately 40–50 meV. These traps are consistent with the trap-assisted SCLC clearly observed in the HRS, dominate conduction at low voltages, and give rise to the temperature-dependent power-law characteristics of the HRS. Nevertheless, their influence on the conduction in the LRS at elevated temperatures above 200 K should not be neglected. (ii) Deep-energy traps, linked to broken CuO chains,^50,51^ are able to trap electrons more robustly due to their greater energy depth. These defects are activated during the SET transition and contribute to the nonvolatility of the LRS by stabilizing hole doping in the CuO_2_ planes. This dual-trap model, comprising point-defect related shallow traps and chain-fragmentation related deep traps, provides a coherent framework for understanding the reversible and robust resistive switching behavior observed in YBCO-based memristors across a wide temperature range.

## Conclusions

We have demonstrated that YBCO-based memristive devices exhibit robust, nonvolatile resistive switching across cryogenic temperatures, governed by trap-controlled space-charge-limited conduction. The switching mechanism is consistent with a field-induced metal–insulator transition, originating from the unique crystal structure and electronic correlations in cuprate superconductors. Our analysis reveals two distinct trap regimes: shallow traps with exponential energy distributions dominate conduction in the HRS, while deep traps—likely associated with CuO chain fragmentation—stabilize the LRS and enable nonvolatile switching. The coexistence of these trap types provides a comprehensive framework for understanding the reversible modulation of electronic phases in YBCO. These findings advance the fundamental understanding of resistive switching in correlated oxides and demonstrate the viability of cuprate superconductors for cryogenic memory and neuromorphic applications. The temperature-independent switching voltages and compatibility with low-temperature operation make these devices promising candidates for integration into superconducting computing architectures and quantum information systems. Importantly, the electrostatic nature of the switching mechanism avoids structural distortion typically associated with electrochemical doping, offering a clean and reversible pathway to modulate electronic phases without the need for ionic conductors or ferroelectric gating layers.

## Experimental Section

### Measurement Routine

Measurements were carried out with a *Keithley Model 2604B* in a *Lake Shore VPF-100* cryostat, with a homemade measurement software. The temperature was controlled with a *Lake Shore Model 335* temperature controller. Temperatures were stabilized within an error of Δ*T* = 0.1 *K* before the voltage pulse trains were executed.

### Device Fabrication

The devices were fabricated starting from bilayers of YBCO and LSMO, which showed superconducting as well as ferromagnetic properties in SQUID-magnetometry. The fabrication method is described in more detail elsewhere.^17^

## Supplementary Material



## Notes

Temperature dependence of *IV* characteristics and resistance hysteresis. Current–voltage characteristics for devices with different areas. Cycle-to-cycle variability. Graphical extraction of the critical voltage. Signs of TAT in the LRS. Power law fitting of intermediate states. (PDF)
