# Supplementary material for: Trap-Controlled Conduction and Metal–Insulator Transition in Superconducting Cuprate Memristors
Source: ACS Appl Electron Mater. 2026 Jan 16;8(3):1099–107. doi: 10.1021/acsaelm.5c02017 (PMC12895420; doi:10.1021/acsaelm.5c02017)
Supplement: Supplementary file 1 [file el5c02017_si_001.pdf]

Supporting Information

Trap-Controlled Conduction and Metal–Insulator Transition in Superconducting Cuprate Memristors

Thomas Günkel[1,2], Enrique Miranda[2], Lluís Balcells[1], Narcís Mestres[1], Anna Palau[1]*, Jordi Suñé[2]*

[1]Insititut de Ciència de Materials de Barcelona, ICMAB-CSIC, Campus de la UAB, 08193 Bellaterra, Spain.
[2]Departament d'Enginyeria Electrònica, Universitat Autònoma de Barcelona, 08193 Bellaterra, Barcelona, Spain

E-mail: palau@icmab.es; jordi.sune@uab.cat

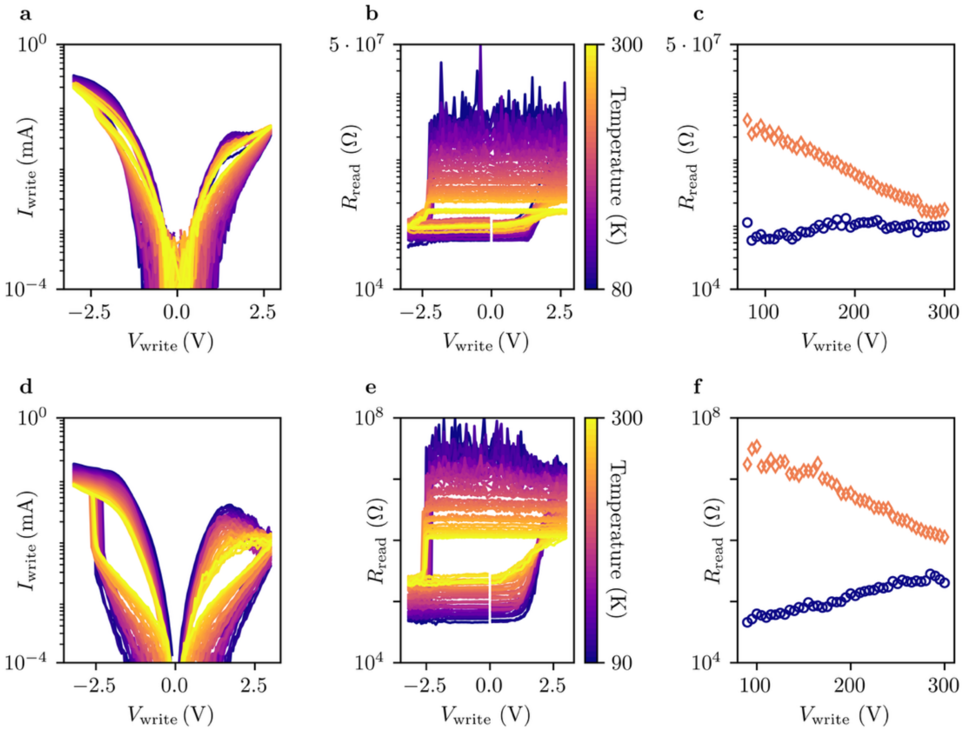

Figure S1: IV-characteristics and resistance hysteresis for a wide temperature range in devices of 50 nm and 100 nm thickness. a,d) IV-characteristics obtained for a) 50 nm device and d) 100 nm device. b,e) Temperature dependence of the resistance hysteresis obtained for the devices shown in a,d. c,f) Extracted HRS (orange diamonds) and LRS (blue circles) from the resistance hysteresis presented in b,e.

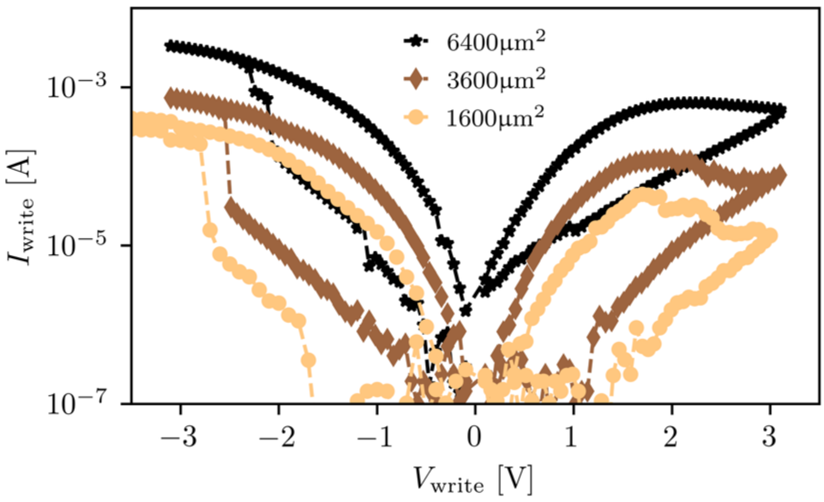

Figure S2: Current-voltage characteristics for devices with different areas. IV hysteresis loops measured for 100 nm YBCO devices, each patterned with different areas as indicated in the legend.

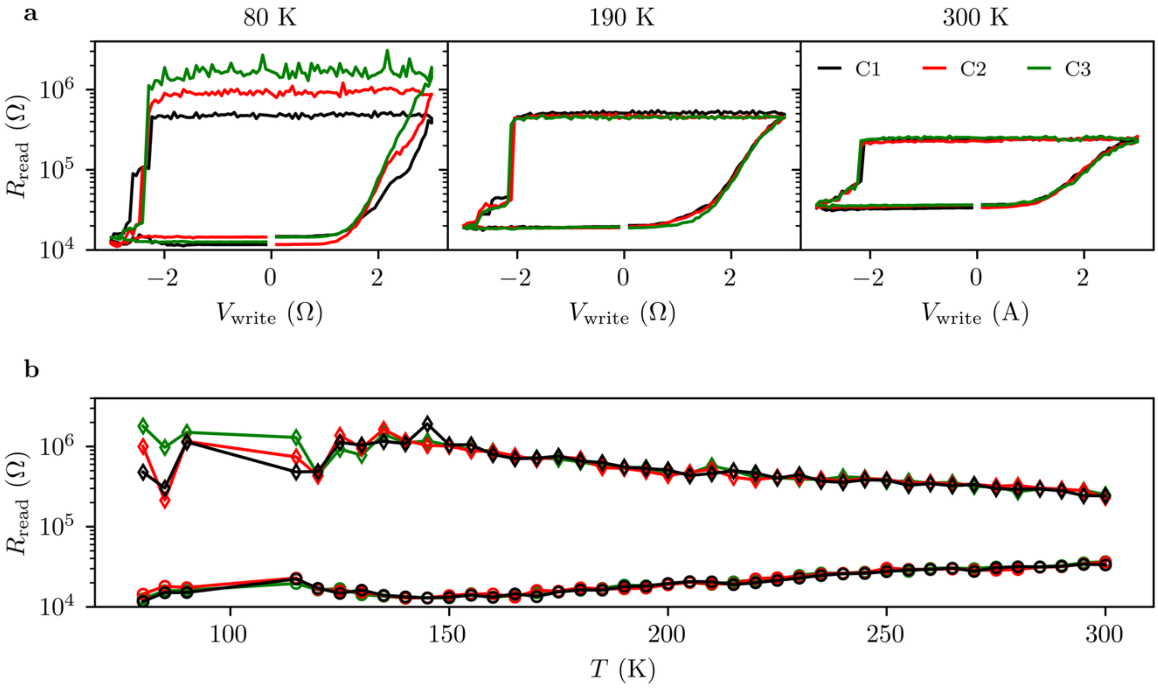

Figure S3: Cycle-to-cycle variability of the device presented in Figure 1 in the main text. a) three cycles for three different temperatures across the whole measurement range (80K-300K). The legend for the subplot at 300 K indicates the cycle number. The legend colour code is valid for all measurements. b) Extracted temperature dependence of the HRS (diamonds) and LRS (circles) obtained using 3 different cycles.

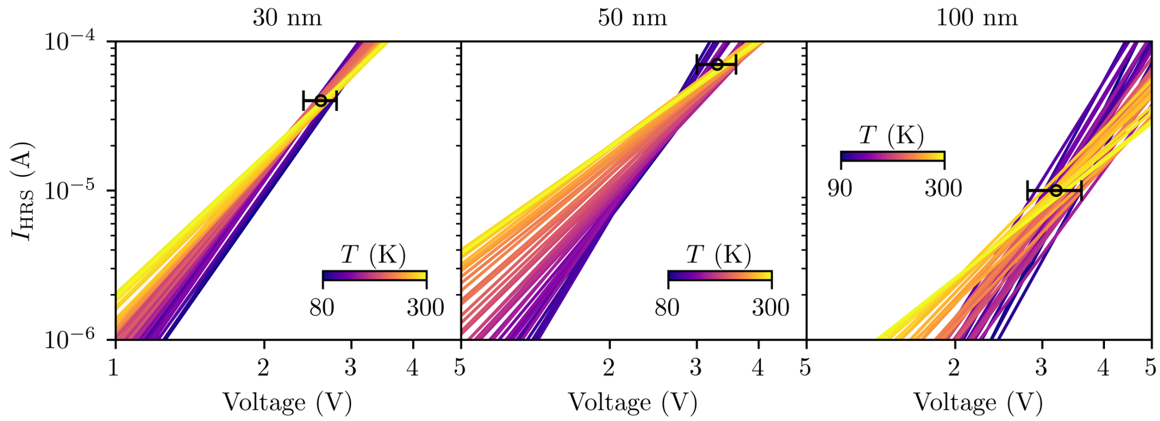

Figure S4: Graphical extraction of the critical Voltage. a) Device of 30 nm thickness, **b)** Device of 50 nm thickness and **c)** Device of 100 nm thickness. Plots show the power law fits for all temperatures indicated in the respective panel. The critical voltage is extracted with a certain error by the intersection of current-voltage characteristics.

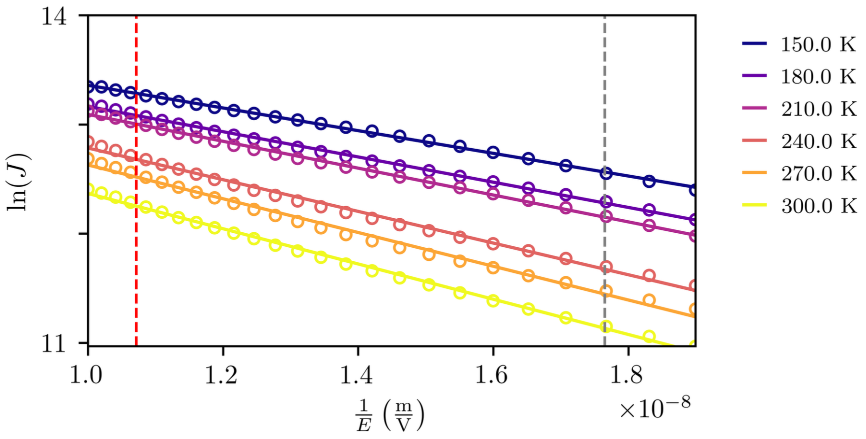

Figure S5: Signs of TAT in the LRS of the 30 nm device. To check if the devices follow the trap assisted tunnelling mechanism in the LRS, the logarithm of the current density is plotted against the inverse of the electric field for various temperatures. To calculate the current density we used the device area of 60x60 $\boldsymbol{\mu m^2}$ and for the electric field a thickness of 30 nm. The red dashed line indicates an applied voltage of 2.8 V and the grey dashed line an applied voltage of 1.7 V. Linearity of these data over a wide voltage range indicate TAT as the governing conduction mechanism in the LRS. The current density derived for the model of trap assisted tunnelling is given by:

$$\boldsymbol{J \propto exp\left(\frac{-8\pi\sqrt{2qm_{eff}}\,\Phi_t^{\frac{3}{2}}}{3h}\frac{1}{E}\right)}$$

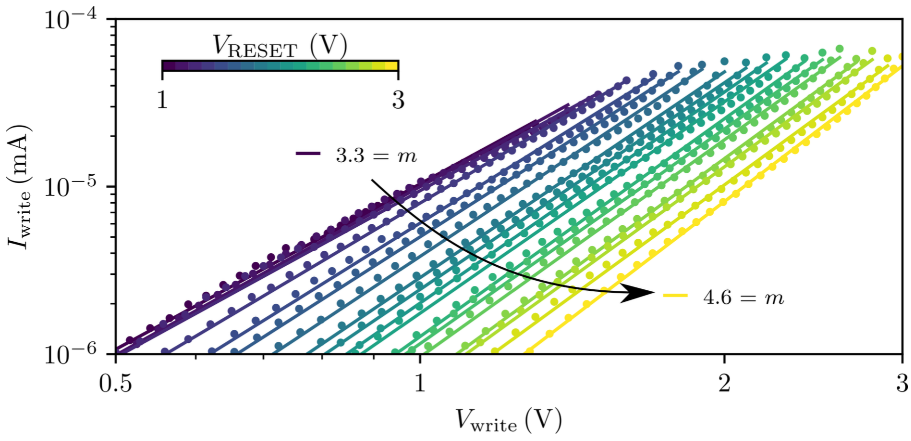

Figure S6: Fitting all intermediate states with a power law dependence. Showing the development of the intermediate states, depending on the applied reset voltage. The reset voltages are given by the colour bar. For increasing reset voltages, a clear trend towards a higher power law exponent is observed. The arrow indicates the transition from the LRS (blue) to the HRS (yellow). Each intermediate state can be reasonably modelled by:

$$\boldsymbol{I \propto V^m}$$
